# Comparison between a New Optical Biometry Device and an Anterior Segment Optical Coherence Tomographer for Measuring Central Corneal Thickness and Anterior Chamber Depth

**DOI:** 10.1155/2016/6347236

**Published:** 2016-06-14

**Authors:** Jinhai Huang, Weicong Lu, Giacomo Savini, Hao Chen, Chengfang Wang, Xinxin Yu, Fangjun Bao, Qinmei Wang

**Affiliations:** ^1^School of Ophthalmology and Optometry, Wenzhou Medical University, Wenzhou, Zhejiang, China; ^2^Key Laboratory of Vision Science, Ministry of Health, Wenzhou, Zhejiang, China; ^3^G.B. Bietti Foundation IRCCS, Rome, Italy

## Abstract

*Purpose.* To compare between a new optical biometer (AL-Scan, Nidek Co., Aichi, Japan) and an anterior segment optical coherence tomographer (Visante AS-OCT, Carl Zeiss Meditec, Dublin, USA) for measuring central corneal thickness (CCT), anterior chamber depth (ACD), and aqueous depth (AD).* Methods.* Sixty-three eyes of 63 normal subjects were examined with AL-Scan and Visante AS-OCT in this prospective study. One eye per subject was measured three times with both devices to record their CCT, ACD, and AD. All procedures were performed by the same operator. Agreement between the two devices was assessed using paired* t*-tests, Bland-Altman plots, and 95% limits of agreement (LoA).* Results.* The mean CCT, ACD, and AD measured by AL-Scan were 538.59 ± 27.37 *μ*m, 3.70 ± 0.30 mm, and 3.16 ± 0.30 mm, respectively. The mean values obtained by the Visante OCT were 536.14 ± 26.61 *μ*m for CCT, 3.71 ± 0.29 mm for ACD, and 3.17 ± 0.29 mm for AD. The mean CCT by the AL-Scan was higher than that obtained by the Visante AS-OCT (difference = 2.45 ± 6.07 *μ*m, *P* < 0.05). The differences in ACD and AD measurements were not statistically significant. The 95% LoA of CCT, ACD, and AD were between −9.44 and 14.35 *μ*m, −0.15 and 0.12 mm, and −0.15 and 0.12 mm, respectively.* Conclusions.* Since these two devices were comparable for measuring CCT, ACD, and AD, their results can be interchangeably used in the clinic.

## 1. Introduction

As cataract and refractive surgery are increasingly performed, the surgeons' skills as well as the precision of ocular measurements are important in order to satisfy patients' expectations. Central corneal thickness (CCT) is critical in designing vision correction surgeries such as laser in situ keratomileusis (LASIK), as well as in glaucoma diagnosis and other corneal diseases [1–6]. Measurements of the anterior chamber depth (ACD), which is defined as the distance from the corneal epithelium to the anterior surface of the crystalline lens, and the aqueous depth (AD), which is defined as the distance from the corneal endothelium to the anterior surface of crystalline lens, have many clinical applications [7]. The ACD measurement is used, for example, by the Holladay 2 formula, whereas the AD measurement is critical for the selection of patients undergoing phakic intraocular lens implantation [8].

Anterior segment optical coherence tomography (Visante AS-OCT, Carl Zeiss Meditec, Dublin, CA, USA) has been clinically used for several years for anterior segment measurement and has high resolution. It is based on low coherence interferometry and uses the light source of a 1310 nm superluminescent light-emitting diode. The Visante AS-OCT is widely used to measure the corneal thickness and ACD. The AL-Scan is a newly introduced optical biometer that can measure six parameters within 10 seconds, including CCT, ACD, axial length (AL), corneal keratometry (K), white-to-white (WTW), and pupil diameter (PD). It uses the principle of the Scheimpflug imaging to measure CCT and ACD and an 830 nm infrared laser diode for AL. Previous studies had reported highly repeatable and reproducible measurements of AL, K values, and ACD with this new device [9–14].

Few studies have investigated the accuracy and agreement of AL-Scan with other instruments [9, 10, 13–15]. This is the first study to compare the ocular measurements obtained by the AL-Scan and the Visante AS-OCT.

## 2. Patients and Methods

Sixty-three eyes of 63 healthy subjects (36 men, 27 women) were enrolled in the study. Mean age (standard deviation, SD) was 23 ± 3.83 years (range: 18–32 years). Mean refraction error was −4.41 ± 2.12 D (range: −0.5 D to −9.00 D). The exclusion criteria were age < 18 years, previous ocular surgery, anterior or posterior pathology, contact lens usage (within 4 weeks for rigid contact lens and within 2 weeks for soft contact lens), and astigmatism > 3.0 D. Before enrolment, each patient underwent a complete ophthalmological examination, including visual acuity, intraocular pressure measurement, anterior segment evaluation, and fundus examination. This study was approved by the Review Board of the Eye Hospital of Wenzhou Medical University and performed according to the Declaration of Helsinki. All patients signed an informed consent document.

AL-Scan uses the principle of the Scheimpflug imaging to measure CCT and ACD with 470 nm monochromatic light emitted from an LED. The anterior chamber single-scan mode was used to measure the CCT and ACD with Visante OCT. The depth and width of the scanning field were 6.0 mm and 16.0 mm, respectively. Scans were centered on the pupil and taken along the horizontal meridian. The scan was obtained when a vertical white line along the center of the cornea was visible. The calibrated caliper function was used to calculate the ACD and AD [16, 17].

All measurements were performed by one experienced examiner. Each subject received three consecutive measurements with the AL-Scan and Visante OCT. We randomly chose only one eye for each patient. All measurements were taken between 10:00 and 17:00 and were completed within 15 minutes for each patient. The measurements were performed in a dimly lit room without pupil dilation.

### 2.1. Statistical Analysis

SPSS software version 21.0 (IBM Corporation, Armonk, NY, USA) and MedCalc Statistical Software V14.8.1 (MedCalc Software, Inc., Belgium) were used for the statistical analysis. The Kolmogorov-Smirnov test was performed to check the data distribution for each device. The paired* t*-test was used to evaluate the difference between the measurements of each device. *P* < 0.05 was considered to be statistically significant. Bland-Altman plots were used to evaluate the differences between the two devices. The range of agreement was shown with 95% limits of agreement (LoA), which stands for the mean difference ± 1.96 SD. Narrower 95% LoA indicated better agreement [18].

## 3. Results

The mean CCT, ACD, and AD measured by AL-Scan were 538.59 ± 27.37 *μ*m, 3.70 ± 0.30 mm, and 3.16 ± 0.30 mm, respectively. The Visante OCT showed 536.14 ± 26.61 *μ*m for CCT, 3.71 ± 0.29 mm for ACD, and 3.17 ± 0.29 mm for AD.

**Figure 1:**
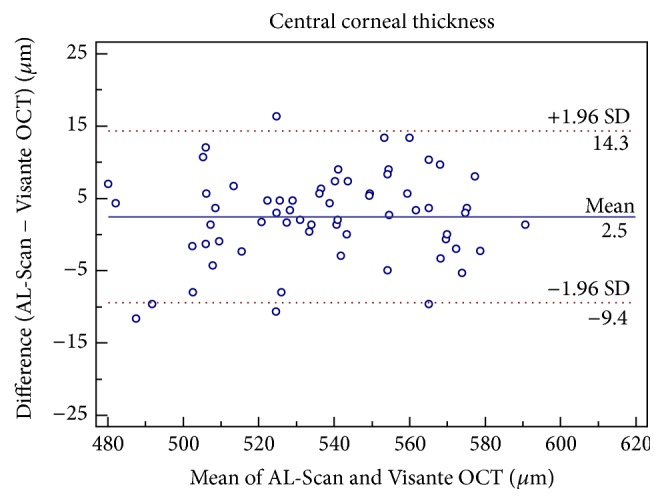
Difference in central corneal thickness measurements between AL-Scan optical biometer and Pentacam rotating Scheimpflug imaging device against their mean values. The solid line indicates the mean difference, and 95% limits of agreement are indicated by solid and dotted lines, respectively.

**Figure 2:**
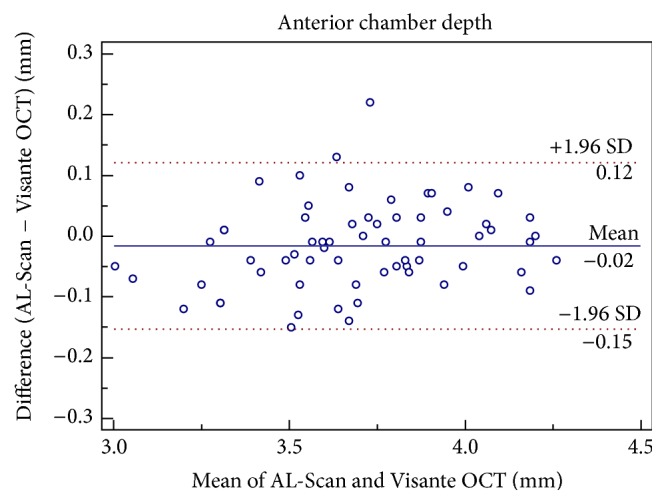
Difference in anterior chamber depth measurements between AL-Scan optical biometer and Pentacam rotating Scheimpflug imaging device against their mean values. The solid line indicates the mean difference, and 95% limits of agreement are indicated by solid and dotted lines, respectively.

**Figure 3:**
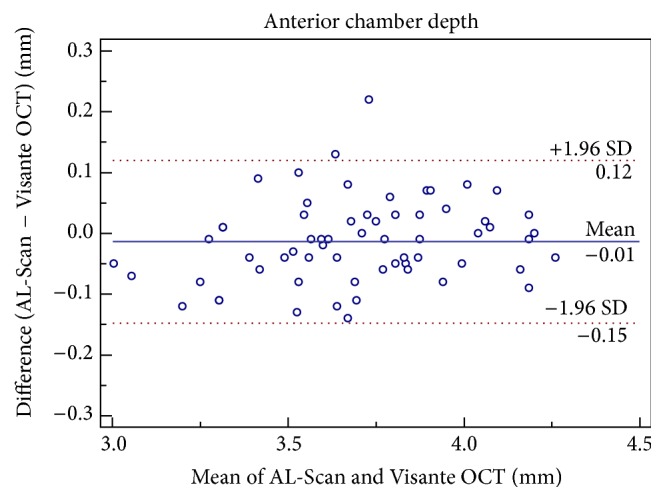
Difference in aqueous depth measurements between AL-Scan optical biometer and Pentacam rotating Scheimpflug imaging device against their mean values. The solid line indicates the mean difference, and 95% limits of agreement are indicated by solid and dotted lines, respectively.

Although there was a statistically significant difference in the mean CCT measurements between the two devices, it was clinically insignificant (Table 1). Good agreement was found between the two devices for CCT with a maximum boundary value of 95% LoA of 14.35 *μ*m (Figure 1). The ACD and AD measurements of AL-Scan and Visante OCT were similar (*P* > 0.05) and had good agreement with the 95% LoA range of −0.15 to 0.12 mm (Figures 2 and 3).

## 4. Discussion

Accurate quantitative measurements of CCT, ACD, and AD provide valuable clinical information and are important for preoperative assessment, surgical planning, and follow-up in phakic IOL implantation. Ultrasound (US) is typically widely used for measuring these parameters [19, 20]. But, nowadays, noncontact devices such as the Visante AS-OCT are more popular in measuring ocular parameters. The AL-Scan is a recently released, noncontact, imaging instrument using partial coherence interferometry (PCI) and the Scheimpflug principle with good repeatability and reproducibility. The Scheimpflug camera with a 470 nm LED is used for measuring the CCT and anterior chamber in the AL-Scan. Our data is the first study to suggest that the Visante AS-OCT and the AL-Scan have good agreement for measuring CCT, ACD, and AD.

Previous studies have investigated the AL-Scan and compared it to other instruments, mainly the IOLMaster. Since the IOLMaster is unable to directly measure the corneal thickness, no prior data for comparing CCT were available. Ethnic variation was found in previous studies when CCT values were measured; Chinese, Caucasians, Hispanics, and Filipinos had comparable CCT measurements, whereas Japanese had significantly thinner corneas than Caucasians, Chinese, Filipinos, and Hispanics. The CCT of African Americans was the thinnest. The differences also existed between the anterior chamber of Chinese and Caucasians [21–23]. So we are more focused on the repeatability results rather than the mean measurement values. Yagci et al. showed high repeatability of CCT values by the AL-Scan in both normal and keratoconic groups. Although its reproducibility was not better than other available Scheimpflug systems, the AL-Scan showed excellent and comparable repeatability and reproducibility in most ocular parameters' measurements [10–12, 24, 25]. Thus, it was useful to review and compare the currently used devices such as Pentacam (Oculus, Wetzlar, Germany), Galilei (Ziemer, Port, Switzerland), and Sirius (Costruzione Strumenti Oftalmici, Florence, Italy) as they all use the principle of the Scheimpflug imaging to measure the CCT despite the lack of direct comparison between the AL-Scan and other Scheimpflug systems. Nam et al. [26] showed that Pentacam can provide comparable and high repeatability of CCT. High CCT repeatability of Sirius was reported by Savini et al. and Huang et al. [27, 28]. A recent analysis showed that the total measurement error of Visante OCT for CCT was 7.88 *μ*m, while the error was 9.85 *μ*m, 7.05 *μ*m, 2.64 *μ*m, and 4.76 *μ*m for ultrasound, Pentacam, Galilei, and Sirius, respectively [29]. Mohamed et al. [30] showed low coefficients of repeatability and reproducibility and high intraclass correlation coefficients of the CCT measurement by Visante OCT. In our recent prospective studies on three different Scheimpflug imaging systems and one OCT, high repeatability and good agreement for CCT measurement were also demonstrated [28, 31]. However, O'Donnell et al. [32] showed that the 95% LoA for Pentacam and Visante OCT were 25.61 to −49.11 *μ*m. In the current study, the Visante OCT provided slightly thinner CCT than AL-Scan, which was also seen between Visante and Pentacam by Nemeth et al. [33], and our max boundary of 95% LoA was 14.35 *μ*m, demonstrating very good agreement between the two devices. The Scheimpflug and OCT measure CCT by different optical and physical techniques: the Pentacam used 475 nm blue light, Visante OCT used 1310 nm diode laser, and AL-Scan used 470 nm LED, which might contribute to the differences in the results [34]. Besides, the anterior corneal surface also influences the demarcating boundary, which results in differences.

A previous study had shown that the total measurement error of IOLMaster for ACD was 0.06 mm and the error of Visante OCT, Pentacam, and Galilei was all approximately 0.05 mm [29]. As compared to the IOLMaster 500, the ACD was 3.17 ± 0.12 mm by AL-Scan and 3.12 ± 0.11 mm by IOLMaster, with a minor difference of 0.13 ± 0.04 mm and high correlation between AL-Scan and IOLMaster 500 in measuring ACD [9]. Srivannaboon et al. [13] also showed a small difference indicating good agreement between AL-Scan and IOLMaster with a LoA range of −0.24 to 0.19 mm, which was similar to the results of our previous comparison between the AL-Scan and IOLMaster [10]. Nemeth et al. [17], Wang et al. [16], and Lavanya et al. [35] showed that the ACD measurements by OCT were 3.11 ± 0.33 mm, 3.76 ± 0.21 mm, and 3.14 ± 0.34 mm, respectively, in normal adults and presented good agreement with ultrasound or Scheimpflug or IOLMaster. Bueno-Gimeno et al. [36] also reported similar results in teenagers. Lavanya et al. [35] demonstrated that ACD measured by Visante OCT had deeper but not clinically important values than IOLMaster. Dinc et al. [37] reported high correlation between Pentacam and Visante OCT in measuring ACD, which was similar in keratoconus in a study by Yazici et al. [34]. In our current study, the 95% LoA range was even narrower for ACD or AD measurements indicating better agreement between AL-Scan and Visante OCT than that between AL-Scan and IOLMaster or between AL-Scan and Galilei [14].

In our current study, three parameters of anterior segment were evaluated. We simultaneously measured ACD and AD modes, which is more comprehensive than other studies that only analyzed one mode. In clinical settings, ACD and the intraocular pressure are important parameters for glaucoma screening and diagnosis. However, the ACD values are the summation of CCT and AD values. Since ACD values can be affected by CCT measurement, the method used for measuring CCT, the accuracy of CCT measurement, corneal edema, and other aspects related to CCT results will influence the precision of ACD. Thus, it was meaningful to assess the agreement of these parameters between the two devices in a single study.

This study had some limitations. We only included healthy unoperated eyes and further investigations are needed to assess both instruments for other categories of patients (such as those affected with keratoconus or previous refractive surgery). Mydriasis would influence changes in the cornea and anterior chamber, so further studies will be performed to evaluate the performance of the biometer after pupil dilation.

This study found a clinically insignificant difference between the two devices for the measurement of CCT. The AL-Scan and Visante AS-OCT have good agreement in measuring CCT, ACD, and AD, and their results can be interchangeably used in the clinical setting.

## Figures and Tables

**Table 1:** Comparison of central corneal thickness (CCT), anterior chamber depth (ACD), and aqueous depth (AD) measured by the AL-Scan partial coherence interferometry and Visante optical coherence tomography.

Device pairings	Mean difference ± SD	*P* value	95% LoA
CCT (*μ*m)	2.45 ± 6.07	0.002	−9.44 to 14.35
ACD (mm)	−0.01 ± 0.07	0.119	−0.15 to 0.12
AD (mm)	−0.02 ± 0.07	0.077	−0.15 to 0.12

SD: standard deviation.
